# The Long-Term Impact of the MEMA kwa Vijana Adolescent Sexual and Reproductive Health Intervention: Effect of Dose and Time since Intervention Exposure

**DOI:** 10.1371/journal.pone.0024866

**Published:** 2011-09-13

**Authors:** Aoife M. Doyle, Helen A. Weiss, Kaballa Maganja, Saidi Kapiga, Sheena McCormack, Deborah Watson-Jones, John Changalucha, Richard J. Hayes, David A. Ross

**Affiliations:** 1 London School of Hygiene & Tropical Medicine, London, United Kingdom; 2 National Institute for Medical Research, Mwanza Centre, Mwanza, Tanzania; 3 Medical Research Council Clinical Trials Unit, London, United Kingdom; Vanderbilt University, United States of America

## Abstract

**Background:**

Despite recent decreases in HIV incidence in many sub-Saharan African countries, there is little evidence that specific behavioural interventions have led to a reduction in HIV among young people. Further and wider-scale decreases in HIV require better understanding of when behaviour change occurs and why. The MEMA kwa Vijana adolescent sexual and reproductive health intervention has been implemented in rural Mwanza, Tanzania since 1999. A long-term evaluation in 2007/8 found that the intervention improved knowledge, attitudes to sex and some reported risk behaviours, but not HIV or HSV2 prevalence. The aim of this paper was to assess the differential impact of the intervention according to gender, age, marital status, number of years of exposure and time since last exposure to the intervention.

**Methods:**

In 2007, a cross-sectional survey was conducted in the 20 trial communities among 13,814 young people (15–30 yrs) who had attended intervention or comparison schools between 1999 and 2002. Outcomes for which the intervention had an impact in 2001 or 2007 were included in this subgroup analysis. Data were analysed using cluster-level methods for stratified cluster-randomised trials, using interaction tests to determine if intervention impact differed by subgroup.

**Results:**

Taking into account multiplicity of testing, concurrence with a priori hypotheses and consistency within the results no strong effect-modifiers emerged. Impact on pregnancy knowledge and reported attitudes to sex increased with years of exposure to high-quality intervention.

**Conclusions:**

The desirable long-term impact of the MEMA kwa Vijana intervention did not vary greatly according to the subgroups examined. This suggests that the intervention can have an impact on a broad cross-section of young people in rural Mwanza.

**Trial registration:**

ClinicalTrials.gov NCT00248469

## Introduction

Young people are at the centre of the HIV pandemic in terms of new infections and opportunities for halting the transmission of HIV[1], [2], [3]. Encouragingly, surveillance data suggests that the UNGASS target of reducing HIV prevalence among young people aged 15–24 yrs living in the most affected countries by 25% by 2010[4] will be met by at least half of the countries where adequate data on trends are available[5]. In many of these countries, declines in HIV prevalence have been accompanied by changes in reported sexual behaviour measured in behavioural surveillance surveys. Nevertheless, there is no direct evidence that specific behaviour change interventions among young people reduce HIV incidence[6], [7]. Furthermore, where surveillance data suggest declines in HIV incidence among young people, decreases have often been recorded only among specific subgroups [5]. Achieving further and wider-scale decreases in HIV requires a better understanding of when behaviour change occurs and why.

One approach to preventing HIV infection is to implement interventions that aim to reduce high-risk sexual behaviours. The MEMA kwa Vijana (MkV) Intervention was one such intervention that has been implemented in rural Mwanza, Tanzania since 1999. The intervention was based on Social Learning Theory and had the following objectives: (i) Delay sexual debut among youth, (ii) Reduce the number of sexual partners among those who are sexually active, (iii) Promote the correct and consistent use of condoms among those who are sexually active, (iv) Increase the uptake of STI and family planning services. The intervention had four main components[8], [9]:

- In-school sexual and reproductive health (SRH) education in years (standards) 5, 6 and 7 of primary schools through a teacher-led, peer-assisted programme.
- Youth-friendly sexual and reproductive health services, through training of the health workers in government health facilities on how to provide attractive and effective SRH services for youth.
- Community-based condom promotion and distribution, for and by youth.
- Community-wide activities to create a supportive environment for adolescent SRH and to begin to address socio-cultural barriers to adolescent behaviour change.

Between 1999 and 2008 the intervention was evaluated within a community randomised trial[9]. Each trial arm comprised 10 communities (∼60 primary schools & ∼ 20 health facilities). An impact evaluation in 2001/2 showed that the intervention led to significant improvements in SRH knowledge, reported attitudes to sex, and some reported behavioural outcomes (age at first sex, number of partners, condom use). Impact tended to be greater among males and improvements in knowledge were greater in unmarried compared to married young people. There was also a trend towards a greater impact on knowledge and reported attitudes in those receiving all 3 years of intervention. There was no consistent impact on biological outcomes.

A long-term (∼9-year) impact evaluation survey was conducted in the same trial communities in 2007/8 involving about 14,000 young people aged 15-30 years who had last been exposed to the in-school component of the intervention on average 5.4 years previously. The intervention had a sustained impact on knowledge (both sexes) and attitudes (males only) and an impact on some but not all of the reported sexual behaviours[10]. However, there was no impact on the primary, biological, outcomes (HIV and HSV2 prevalence)[10]. These results and those of similar studies suggest that additional interventions targeting the broader community and/or structural factors are needed[7], [10], [11]. However, interventions such as MEMA kwa Vijana will remain an important component of the HIV prevention package because knowledge is often a pre-requisite for effective behaviour change. As such, it is essential to obtain a more detailed understanding of the patterns of intervention impact on knowledge and other outcomes.

In this paper we analyse details of the intervention impact, including the effect of receiving more years of the intervention, possible attenuation of intervention impact over time, and variation in intervention effect by other factors (age/marital status). Given the need for additional interventions (see above) one important question is whether the intensity/duration of a school-based intervention can be reduced.

## Methods

### Ethics statement

The trial protocol received ethical clearance from the Tanzanian Medical Research Coordinating Committee and the Ethics Committee of the London School of Hygiene & Tropical Medicine. Signed informed consent was obtained from each participant on the day of the survey round. Additional signed consent was obtained from parents of participants under the age of 18 years.

### Data collection

Full study details have been published previously[10]. Briefly, between June 2007 and July 2008 a household census was conducted in the 20 MkV trial communities in rural Mwanza. All young people aged 15–30 years who were thought to have attended primary school years 5–7 of the intervention (or comparison) schools between 1999 and 2002 were invited to a survey at a central location in their village. At the survey site, eligible attendees who gave informed consent were interviewed about their knowledge, attitudes, and reported sexual behaviour. Blood and urine specimens were collected. A clinician asked about STI symptoms and examined males for circumcision and for signs of STIs. HIV counselling and rapid testing were offered.

### Laboratory analysis

Sera were tested for antibodies to HSV-2 using Kalon HSV Type 2 IgG ELISA (Kalon Biologicals, Guildford, UK) following the manufacturer's instructions. KALON ELISA indeterminate samples were retested. Persistently indeterminate specimens were classified as negative. Other laboratory assays performed for this survey included HIV ELISA and PCR for gonorrhea and *Chlamydia trachomatis.*

### Study design

The intervention started in 1999, was implemented in standards 5–7 of primary school and was phased in over a 3 year period. In 2003, teacher training and supervision of teachers and health facility staff were reduced and monitoring suggests that the quality, fidelity and coverage of the intervention decreased from this time (Ross DA, personal communication). Two measures of “intervention exposure” were, therefore, considered:

*‘Total intervention exposure’* - number of years of exposure between 1999 and 2004 (all eligible participants are assumed to have left primary school by the end of 2004)

*‘High-quality intervention exposure’* - number of years of exposure between 1999 and 2002.

**Figure 1:**
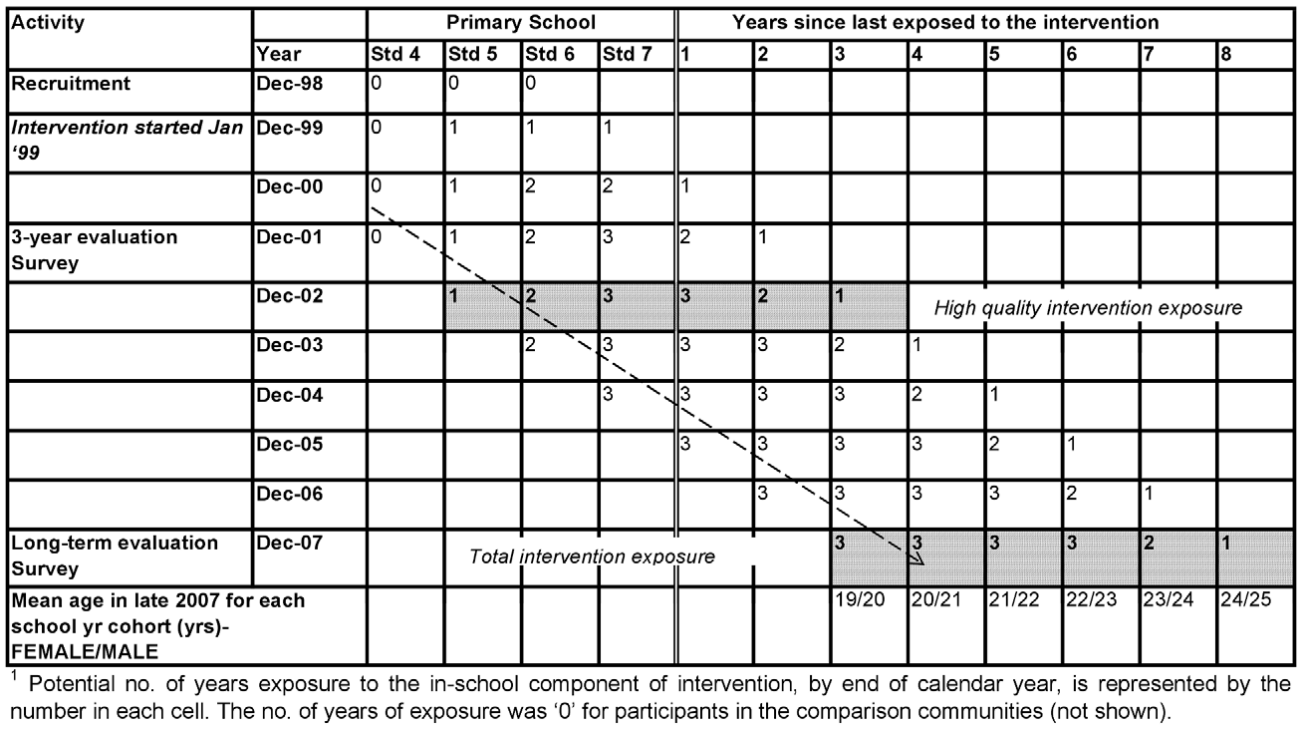
Cohort diagram showing those eligible for the 2007/8 MEMA kwa Vijana impact evaluation survey^1^.

Figure 1 shows the flow of participants exposed to the in-school component of the MkV intervention. The columns represent the years in primary school (exposure to the intervention) and the number of years since leaving primary school. The mean age of each school year cohort is given in the final row. The total number of years of intervention exposure is given by the number in each cell. For example, the dashed arrow indicates that those in standard 4 in 2000 had 2 years of high-quality intervention exposure by the end of 2002. By the end of 2007 they had 3 total years of intervention exposure, 2 years of which were high-quality, and it was four years since their last exposure to the in-school component of the intervention.

### Hypothesised effect modifiers

The subgroup analyses were planned prior to the long-term impact evaluation of the intervention and were based on the following a priori hypotheses:

1. The impact of the intervention would differ by gender of the participant. For example, male participants may have had a greater ability to make decisions about their sex lives.
2. The impact of the intervention would differ by age. We hypothesised greater impact among older participants (in 2007/8) as they might have been more empowered and able to change their behaviour[12].
3. The intervention impact would differ by marital status in 2007/8. For example, unmarried participants may have had greater scope to change their risk-taking behaviours such as condom use[13], [14], [15].
4. The intervention impact would differ by years of exposure to the intervention. It was further hypothesised that a dose-response effect, if one existed, would be more likely to be seen when looking at duration of *High-quality* intervention exposure.
5. The intervention would have had a greater impact on those exposed to the intervention in the more recent past.

### Outcomes

The MkV trial collected data on the following outcomes: SRH knowledge (HIV acquisition, STI acquisition, pregnancy prevention), attitudes to sex, reported behaviour (sexual debut, number of partners, condom use, contraceptive use, use of health facility for recent STI symptoms) and biological outcomes (HIV, HSV2, Syphilis, *Chlamydia trachomatis*, gonorrhea, pregnancy, genital discharge, genital ulcer). To limit the possibility of detecting spurious effects, only outcomes on which the intervention was shown to have had a statistically significant (p<0.05) impact in either 2001/2 or in 2007/8 were included i.e. all knowledge outcomes, reported attitudes and selected reported behaviours (age at first sex; number of partners; condom use)[9], [10]. To ensure adequate power to detect a 40% difference in intervention impact, analyses were restricted to outcomes with an overall prevalence > 10% when data were stratified by age group/marital status/high -quality dose, and > 20% when stratified by total dose/years since last exposure to intervention. The following outcomes were thus excluded: condom use at last sex in the last 12 months with a non-regular partner, genital ulcer syndrome, gonorrhoea prevalence. For completeness, the more prevalent primary outcome (HSV2 prevalence), was included even though there was no overall intervention impact on HSV2. The study did not have enough power to detect any subgroup effects for the other primary outcome (HIV prevalence).

### Statistical analysis

Details of allocation to trial arm and sample size calculations are provided elsewhere[8], [9], [10]. The data were analysed using cluster-level methods for stratified cluster-randomised trials[16]. For each outcome, the unadjusted prevalence ratio was calculated as the ratio of the geometric mean prevalence for the ten communities in each arm, or the ratio of arithmetic mean prevalence if the outcome had zero cases in at least one community. Adjusted prevalence ratios (aPRs) were calculated as the geometric or arithmetic mean ratio of observed to expected prevalence(O/E) with logistic regression used to estimate the expected prevalence, adjusted for individual-level covariates (see footnotes to tables) 95% confidence intervals (CI) for the adjusted prevalence ratios were estimated using the residual mean square from a two-way analysis of variance (ANOVA) of community log (O/E) on stratum and study arm with 14 degrees of freedom[16]. The adjusted prevalence ratios within levels of each subgroup were estimated in the same way, but stratified by level of the subgroup.

Interaction tests were used to analyse subgroup effects. For gender and marital status, a stratified t-test was used to compare the difference in log-prevalence between arms[17]. For age, years of exposure and time since exposure, Cheung's method was extended by using linear regression to estimate the dose-response for each community, and by conducting a t-test to compare the regression coefficients between trial arms[17]. In order to minimise over–interpretation of the results, the findings are discussed in terms of whether the subgroup effect is ‘highly plausible’ to ‘extremely unlikely’ depending on the size of the p-value and the consistency across related outcomes[18]. We considered the plausibility of all subgroup effects where p≤0.20. Stata Version 11.0 (Stata Corp, College Station, Texas, USA) was used for all analyses.

## Results

The household census and follow-up visits to nearby secondary schools and major migration points identified 16,747 young people who were potentially eligible to participate in the MkV long-term impact evaluation survey. 13,281 (79%) of invited individuals attended on the survey day along with 2,426 non-invited young people. A total of 13,814 (88%) out of the 15,707 young people who attended the survey were deemed eligible to participate including 3,808 (40%) of the original trial cohort[10].

The median age for males and females was 22 years and 21 years respectively (Table 1). The majority of participants were from the Sukuma ethnic group (80%). Over half the females and one third of the males were married. 92% reported ever having had sex and the median reported age at sexual debut was 18 years among intervention males and 17 years among comparison males and among females in both trial arms (Table 1).

**Table 1:** Characteristics of the 13,814 Long-term Evaluation (2007/8) participants, by sex and trial arm.

Variable	Male (n = 7300)		Female (n = 6514)	
	Intervention	Comparison	Intervention	Comparison
	N = 3807	N = 3493	N = 3276	N = 3238
**Age (years)**				
<20	660 (17%)	503 (14%)	868 (27%)	795 (25%)
20-21	1005 (26%)	906 (26%)	953 (29%)	1001 (31%)
22-23	1017 (27%)	1010 (29%)	860 (26%)	909 (28%)
> = 24	1124 (30%)	1074 (31%)	594 (18%)	532 (16%)
**Median Age (years)**	22	22	21	21
**Ethnic Group (Sukuma)**	2882 (76%)	2834 (81%)	2549 (78%)	2747 (85%)
**Religion**				
Christian	3099 (81%)	2784 (80%)	2860 (87%)	2905 (90%)
Muslim	143 (4%)	187 (5%)	142 (4%)	136 (4%)
Other religion	20 (0.5%)	38 (1%)	7 (0.2%)	2 (0.1%)
None	542 (14%)	476 (14%)	260 (8%)	187 (6%)
**Currently married**	1242 (33%)	1202 (34%)	1806 (55%)	1858 (57%)
**Ever married**	1346 (35%)	1327 (38%)	2121 (65%)	2168 (67%)
**Highest level of education**				
2° school or higher	864 (23%)	678 (19%)	472 (14%)	411 (13%)
**Male circumcision (clinical examination)**	1596 (43%)	1315 (38%)	NA	NA
**Median reported age at sexual debut (years)**	18	17	17	17
**Years of exposure to in-school component of MEMA kwa Vijana between 1999 and 2004 (total intervention exposure)** 1				
1 year	629 (17%)	576 (16%)	515 (16%)	517 (16%)
2 years	616 (16%)	647 (19%)	555 (17%)	518 (16%)
3 or more years	2562 (67%)	2270 (65%)	2206 (67%)	2203 (68%)
**Years of exposure to in-school component of MEMA kwa Vijana between 1999 and 2002 (high quality intervention exposure)** 1				
1 year	1358 (36%)	1136 (33%)	1156 (35%)	1157 (36%)
2 years	1241 (33%)	1159 (33%)	1065 (33%)	980 (30%)
3 or more years	1208 (32%)	1198 (34%)	1055 (32%)	1101 (34%)
**Years since last exposure to in-school intervention** 1				
3-4 yrs	1426 (37%)	1117 (32%)	1208 (37%)	1144 (35%)
5-6 yrs	1245 (33%)	1234 (35%)	1097 (33%)	1129 (35%)
7-8 yrs	1136 (30%)	1142 (33%)	971 (30%)	965 (30%)
**Mean number of years**	5.4	5.5	5.4	5.4

1 or exposure to equivalent years in comparison school.

Two-thirds of respondents had a total exposure of at least 3 years of the in-school component of the intervention but only one-third had 3 years of high-quality exposure. Participants had, on average, been last exposed to the in-school component of the intervention (or comparison) 5.4 years prior to the survey (Table 1).

### Impact according to gender

There was no evidence of differential intervention impact according to the gender of the participant (Table 2) and subsequent results are, therefore, not presented stratified by gender.

**Table 2:** Impact of intervention on selected primary and secondary outcomes according to gender 1.

Outcome	Overall	Male	Female	p-value^2^
**HIV acquisition knowledge**(% with all 3 responses “correct”)	**1.11 (1.01, 1.23)**	1.11 (0.99, 1.23)	1.11 (1.00, 1.24)	0.81
**STD acquisition knowledge**(% with all 3 responses “correct”)	**1.20 (1.04, 1.39)**	1.18 (1.04, 1.34)	1.24 (0.97, 1.58)	0.58
**Pregnancy prevention knowledge**(% with all 3 responses “correct”)	**1.18 (1.10, 1.26)**	1.19 (1.12,1.26)	1.17 (1.06,1.30)	0.68
**Reported attitudes to sex**(% with all 3 responses “correct”)	**1.23 (0.94, 1.63)**	1.31 (0.97,1.77)	1.09 (0.67,1.77)	0.42
**Age at first sex < 16y**	**0.96 (0.85, 1.09)**	0.91 (0.80,1.05)	1.01 (0.80,1.28)	0.40
**>2 (female) or >4 (male) lifetime sexual partners**	**0.88 (0.78, 0.99)**	0.87 (0.78,0.97)	0.89 (0.75,1.05)	0.74
**>1 partner in last 12 m**	**0.93 (0.82, 1.07)**	0.92 (0.79,1.08)	0.97 (0.76,1.23)	0.74
**Used condom at last sex in past 12m** 3	**1.22 (0.97, 1.53)**	1.19 (0.91,1.54)	1.27 (0.97,1.67)	0.53
**HSV-2 prevalence**	**0.96 (0.85, 1.09)**	0.94 (0.77,1.15)	0.96 (0.87,1.06)	0.79

1.Prevalence ratio adjusted for age group, stratum and ethnic group (Sukuma vs non-Sukuma).

2. From test for interaction.

3. Among those who reported having had sex in past 12m.

### Impact according to age group in 2007/8

Overall there was little evidence that intervention impact varied by age-group at the 2007/8 survey (Table 3). There was weak evidence that the intervention led to a reduction in the reported number of sexual partners in the last 12 months among the three older age groups and an increase of reporting of sexual partners among the youngest age group (p = 0.03). There was some suggestion that the intervention impact on HSV2 prevalence varied according to age group though no clear trend was seen and the evidence for such an interaction was very weak (p = 0.13).

**Table 3:** Impact of intervention on selected primary and secondary outcomes according to age group in 2007/8^1^.

Outcome	Overall	<20 yrs	20–21 yrs	22–23 yrs	24+ yrs	p-value^2^
**HIV acquisition knowledge**(% with all 3 responses “correct”)	**1.11 (1.01,1.23)**	1.07 (0.97, 1.18)	1.10 (0.99, 1.23)	1.15 (1.01, 1.30)	1.11 (1.00, 1.23)	0.28
**STD acquisition knowledge**(% with all 3 responses “correct”)	**1.20 (1.04,1.39)**	1.16 (0.96, 1.41)	1.26 (1.08, 1.48)	1.22 (1.01, 1.46)	1.16 (0.99, 1.37)	0.91
**Pregnancy prevention knowledge**(% with all 3 responses “correct”)	**1.18 (1.10,1.26)**	1.17 (1.06, 1.28)	1.17 (1.06, 1.29)	1.20 (1.10, 1.30)	1.20 (1.13, 1.28)	0.46
**Reported attitudes to sex**(% with all 3 responses “correct”)	**1.23 (0.94,1.63)**	1.13 (0.82, 1.54)	1.22 (0.91, 1.64)	1.28 (0.90, 1.83)	1.24 (0.92, 1.67)	0.39
**Age at first sex <16y**	**0.96 (0.85,1.09)**	0.93 (0.75, 1.15)	1.03 (0.90, 1.18)	0.92 (0.77, 1.09)	1.00 (0.84, 1.19)	0.72
**>2 (female) or >4 (male) lifetime sexual partners**	**0.88 (0.78,0.99)**	0.98 (0.77, 1.26)	0.81 (0.70, 0.94)	0.85 (0.76, 0.95)	0.93 (0.82, 1.04)	0.70
**>1 partner in last 12 months**	**0.93 (0.82,1.07)**	1.19 (0.91, 1.55)	0.88 (0.74, 1.05)	0.90 (0.76, 1.06)	0.90 (0.80, 1.02)	0.03
**Used condom at last sex in past 12m** 3 **^,^** 4	**1.22 (0.97,1.53)**	1.17 (0.93, 1.47)	1.18 (0.90, 1.55)	1.27 (0.96, 1.67)	1.17 (0.86, 1.60)	0.84
**HSV-2 prevalence**	**0.96 (0.85,1.09)**	1.12 (0.89, 1.41)	0.90 (0.75, 1.09)	0.98 (0.87, 1.10)	0.94 (0.84, 1.06)	0.13

1. Overall prevalence ratio adjusted for gender, age group, stratum and ethnic group (Sukuma vs non-Sukuma). Prevalence ratio for each age group adjusted for gender, stratum and ethnic group (Sukuma vs non-Sukuma).

2. From test for interaction.

3. Among those who reported having had sex in past 12m.

4. Analysis using arithmetic means.

### Impact according to marital status

There was no evidence of effect modification by marital status (Table 4).

**Table 4:** Impact of intervention on selected primary and secondary outcomes according to marital status in 2007/81.

Outcome	Overall	Currently married	Not currently married	p-value^2^
**HIV acquisition knowledge**(% with all 3 responses “correct”)	**1.11 (1.01,1.23)**	1.13 (1.00,1.28)	1.10 (1.01,1.20)	0.42
**STD acquisition knowledge**(% with all 3 responses “correct”)	**1.20 (1.04,1.39)**	1.18 (0.97,1.44)	1.22 (1.05,1.41)	0.68
**Pregnancy prevention knowledge**(% with all 3 responses “correct”)	**1.18 (1.10,1.26)**	1.20 (1.09,1.31)	1.18 (1.09,1.27)	0.70
**Reported attitudes to sex**(% with all 3 responses “correct”)	**1.23 (0.94,1.63)**	1.27 (0.91,1,78)	1.19 (0.91,1.54)	0.54
**Age at first sex < 16y**	**0.96 (0.85,1.09)**	0.97 (0.83,1.14)	0.97 (0.83,1.13)	0.95
**>2 (female) or >4 (male) lifetime sexual partners**	**0.88 (0.78,0.99)**	0.88 (0.77,1.00)	0.89 (0.79,1.01)	0.76
**>1 partner in last 12 m**	**0.93 (0.82,1.07)**	0.93 (0.82,1.05)	0.93 (0.79,1.10)	0.94
**Used condom at last sex in past 12m** 3	**1.22 (0.97,1.53)**	1.15 (0.77,1.72)	1.16 (0.98,1.38)	0.97
**HSV-2 prevalence**	**0.96 (0.85,1.09)**	0.97 (0.87,1.08)	0.98 (0.84,1.15)	0.75

1.Prevalence ratio adjusted for gender, age group, stratum and ethnic group (Sukuma vs non-Sukuma).

2. From test for interaction.

3. Among those who reported having had sex in past 12m.

### Impact according to years of intervention exposure

There was little evidence of a dose-response effect when total intervention exposure was considered (data not shown). A dose-response was slightly more evident when analyses were stratified by high-quality intervention exposure (Table 5). In particular, there was strong evidence that the impact on pregnancy prevention knowledge (p = 0.005) and reported attitudes to sex (p = 0.008) increased with increasing high-quality intervention exposure. There was also very weak evidence that reported use of condom at last sex increased with increasing high-quality intervention exposure (p = 0.19).

**Table 5:** Impact of intervention on selected primary and secondary outcomes in 2007/8 according to number of years of exposure to ‘High Quality’ in-school intervention (1999-2002)1.

	Overall	Yrs of in-school intervention (99-02)			
Outcome		1 yr	2 yrs	3+ yrs	p-value^2^
**Knowledge**					
**HIV acquisition knowledge**(% with all 3 responses “correct”)	**1.11 (1.01,1.23)**	1.11 (1.00, 1.22)	1.12 (0.99, 1.27)	1.12 (0.98, 1.27)	0.78
**STD acquisition knowledge**(% with all 3 responses “correct”)	**1.20 (1.04,1.39)**	1.20 (1.04, 1.38)	1.21 (1.02, 1.42)	1.21 (1.02, 1.44)	0.85
**Pregnancy prevention knowledge**(% with all 3 responses “correct”)	**1.18 (1.10,1.26)**	1.13 (1.04, 1.22)	1.20 (1.12, 1.29)	1.23 (1.13, 1.33)	0.005
**Reported attitudes to sex**(% with all 3 responses “correct”)	**1.23 (0.94,1.63)**	1.11 (0.81, 1.52)	1.21 (0.93, 1.57)	1.41 (1.05, 1.88)	0.008
**Age at first sex <16y**	**0.96 (0.85,1.09)**	0.98 (0.86, 1.12)	0.97 (0.86, 1.11)	0.95 (0.79, 1.15)	0.65
**>2 (female) or >4 (male) lifetime sexual partners**	**0.88 (0.78,0.99)**	0.90 (0.77, 1.04)	0.93 (0.82, 1.05)	0.83 (0.71, 0.97)	0.28
**>1 partner in last 12 months**	**0.93 (0.82,1.07)**	0.99 (0.82, 1.19)	0.92 (0.78, 1.10)	0.91 (0.79, 1.06)	0.34
**Used condom at last sex in past 12m** 3	**1.22 (0.97,1.53)**	1.19 (0.95, 1.49)	1.13 (0.91, 1.40)	1.33 (0.98, 1.82)	0.19
**HSV-2 prevalence**	**0.96 (0.85,1.09)**	0.98 (0.84, 1.15)	0.94 (0.84, 1.05)	0.98 (0.83, 1.14)	0.92

1 Overall prevalence ratio adjusted for gender, age group, stratum and ethnic group (Sukuma vs non-Sukuma). Prevalence ratio according to dose adjusted for gender, age group, stratum and ethnic group (Sukuma vs non-Sukuma) and years since exposure to the in-school component of the intervention.

2. From test for interaction.

3. Among those who reported having had sex in past 12m.

### Impact according to time since last exposure to the intervention

There was little evidence that the impact varied according to the number of years since respondents were last exposed to the in-school component of the intervention (Table 6). There was very weak evidence of a greater intervention impact on condom use at last sex in the last 12 months among those who were exposed to the intervention in the more distant past (p = 0.15).

**Table 6:** Impact of intervention on selected primary and secondary outcomes in 2007/8 according to years since last exposure to in-school intervention^1^.

	Overall	Yrs since exposure to the in-school intervention			
Outcome		3–4 yrs	5–6 yrs	7–8 yrs	p-value^2^
**HIV acquisition knowledge**(% with all 3 responses “correct”)	**1.11 (1.01,1.23)**	1.12 (1.01, 1.24)	1.12 (0.98, 1.28)	1.11 (1.01, 1.22)	0.83
**STD acquisition knowledge**(% with all 3 responses “correct”)	**1.20 (1.04,1.39)**	1.20 (1.00, 1.42)	1.23 (1.04, 1.45)	1.19 (1.01, 1.40)	0.92
**Pregnancy prevention knowledge** (% with all 3 responses “correct”)	**1.18 (1.10,1.26)**	1.17 (1.08, 1.27)	1.22 (1.12, 1.34)	1.16 (1.08, 1.25)	0.88
**Reported attitudes to sex**(% with all 3 responses “correct”)	**1.23 (0.94,1.63)**	1.14 (0.86, 1.51)	1.38 (1.02, 1.88)	1.19 (0.88, 1.61)	0.60
**Age at first sex <16y**	**0.96 (0.85,1.09)**	0.98 (0.86, 1.11)	0.98 (0.82, 1.17)	0.95 (0.84, 1.08)	0.67
**>2 (female) or >4 (male) lifetime sexual partners**	**0.88 (0.78,0.99)**	0.93 (0.76, 1.13)	0.84 (0.72, 0.98)	0.90 (0.82, 0.99)	0.72
**>1 partner in last 12 months**	**0.93 (0.82,1.07)**	0.99 (0.78, 1.24)	0.92 (0.79, 1.07)	0.91 (0.80, 1.04)	0.39
**Used condom at last sex in past 12m** 3	**1.22 (0.97,1.53)**	1.08 (0.89, 1.31)	1.34 (0.97, 1.84)	1.28 (0.96, 1.70)	0.15
**HSV-2 prevalence**	**0.96 (0.85,1.09)**	0.99 (0.83, 1.17)	0.98 (0.83, 1.15)	0.94 (0.86, 1.03)	0.33

1 Overall prevalence ratio adjusted for gender, age group, stratum and ethnic group (Sukuma vs non-Sukuma). Prevalence ratio according to years since exposure to the intervention adjusted for gender, age group, stratum and ethnic group (Sukuma vs non-Sukuma) and total years of exposure to the in-school component of the intervention (99-04).

2. From test for interaction.

3. Among those who reported having had sex in past 12m.

## Discussion

The MkV intervention had a long-term impact on a number of knowledge, attitude and reported sexual behaviour outcomes[10]. This paper presents intervention impact evaluation results for pre-defined population subgroups: age and marital status at time of the survey, years since exposure and number of years of exposure to the in-school component of the intervention (high-quality exposure (1999–2002) and total exposure (1999–2004)). Overall there was little variation of intervention impact according to the subgroups examined. There was no significant variation in intervention impact on the HIV/STD knowledge outcomes. A strong dose-response effect was seen for pregnancy prevention knowledge and reported attitudes to sex when years of the predefined high-quality intervention exposure were considered. There was some evidence that intervention impact on reported number of sexual partners in the last 12 months and HSV2 prevalence differed according to age group, and that reported use of condom at last sex was highest among those who had the greatest exposure to the intervention and among those who were exposed to the intervention in the most distant past.

We predicted that the intervention impact would increase with age of the participant at the time of the long-term impact survey but this pattern was seen only for reported number of recent sexual partners. However, older participants were more likely to have been exposed to the intervention in the more distant past (Figure 1) and less likely to have received the full 3 years of the intervention, and the effect modification by age may have been partially or totally confounded by time since exposure and number of years of exposure to the intervention. We also predicted that the intervention would have the greatest impact among unmarried young people but we found little evidence to support this hypothesis.

Of key interest to intervention programmers and implementers is the dose of intervention required to have an impact. In the 2001/2 follow-up survey, three years after the intervention commenced, there was strong evidence that a greater number of years of the in-school component of the intervention was associated with a larger impact for outcomes that had been significantly affected by the intervention[9]. This trend was strongest for male participants[9]. The 2007/8 follow-up survey data presented in this paper also provide some evidence of a dose-response effect, yet this was not present for all outcomes. A dose-response effect was particularly evident for the pregnancy prevention knowledge and reported attitudes to sex outcome and this is consistent with the 2001/2 evaluation where there was also strong evidence of a dose-response effect for these outcomes [9]. As predicted there was greater evidence of an intervention dose-response effect when exposure to the intervention between 1999 and 2002 (high-quality intervention exposure) was considered. This supports the notion that, in the absence of supervision of intervention staff and regular refresher and replacement training for teachers, the quality and intensity and hence impact of the intervention are likely to have decreased.

We hypothesised that the impact of the intervention may have waned as time since exposure to the intervention teachings increased. Contrary to our initial hypothesis, we found very weak evidence that for the condom use at last sex outcome the intervention impact was greatest among those who were exposed during the earlier years of the intervention i.e. in the most distant past. It is important to note that these analyses were adjusted for age in 2007/8 and total intervention exposure (99-04). Adjusting instead for years of high-quality intervention exposure (99-02) did not change the above results. Age, years of high-quality intervention exposure and years since exposure to the intervention are all closely related and it was difficult to separate out the independent effects of each of these potential effect modifiers.

When compared to the 2001/2 (3-year) evaluation survey the 2007/8 (9-year) survey found evidence of an impact on fewer outcomes and evidence of a smaller impact on those outcomes. For example, in 2001/2 there was strong evidence of a substantial impact of the intervention on the composite knowledge and attitude outcomes with the adjusted risk ratios for these four outcomes ranging from 1.28 to 1.77 for male and from 1.41 to 1.58 in female participants. In 2007/8, there was only borderline to moderate evidence of an impact on these outcomes with adjusted prevalence ratios ranging from 1.11 to 1.31 for males and from 1.11 to 1.24 for female participants. However, a direct comparison between the 2001/2 and 2007/8 results is not valid as the 2007/8 sample contained only a proportion of the original 2001/2 cohort and it is likely that the quality and intensity of the intervention may have varied over time. In general, the decrease in overall effect between the two follow-up surveys may have made it more difficult to detect any subgroup effects.

A major strength of this study was the large sample size and the availability of data on the long-term impact of the intervention. The randomised design helped to ensure that there were no systematic differences, known or unknown, between trial arms that would have affected the outcomes. In order to minimise chance findings, the subgroup analyses were planned *a priori.* We analysed 45 outcomes in total (9 within each sub-group), so we would expect 2 associations with p<0.05 due to chance alone. As recommended [18], [19], [20], therefore, the results were interpreted with caution, taking into account not only the strength of evidence but the consistency within the data and concurrence with a priori hypothesis. Subgroup analysis was not powered to see a difference in the primary outcome (HIV prevalence) nor some of the other less prevalent outcomes e.g. genital ulcer syndrome. However, there was little overall intervention impact on those outcomes and substantial subgroup effects were therefore unlikely. The study was only powered to detect large subgroup effects and as such some true interactions may not have been detected.

The results of this study depend on the validity of the measures of the study outcomes and potential effect modifiers. As with many studies that are based on reported behavioural data, we cannot exclude the possibility of reporting bias. Such bias would be particularly important if levels of under or over-reporting varied according to intervention status and/or by the potential effect modifier. Some degree of differential reporting bias is likely although, given the age of the respondents and the relatively long time since exposure to the intervention, we think that this would have been minimal. Intervention exposure and years since exposure to the intervention were calculated based on a set of detailed questions relating to years attended school. While we do not suspect that recall would have varied between trial arm, it is possible that incorrect recall led to a masking of effects or observation of spurious effects. Attending school during a certain year is a crude measure of real exposure to the intervention which was dependant on attendance at school and, appropriate delivery of the curriculum by the teachers. Only exposure to the in-school component of this multi-component intervention was considered but this was the largest and believed to have been the most influential component of the intervention. Retrospective measurement of exposure to the other components (use of health facilities, contact with condom distributors and participation in community activities) would have been even more problematic. Despite considerable effort to trace young people eligible to participate in the survey, selection bias may have occurred if certain subgroups of young people, such as the more mobile, were less likely to participate. Such bias is unlikely to have differed between trial arms. If any of the above biases varied according to the subgroups examined then this should not have biased the estimate of effect within each subgroup but may have decreased the power of the study to detect differences between the subgroups.

A recent systematic review of HIV prevention interventions among young people in sub-Saharan Africa[7] found that few studies had appropriately evaluated dose-response effects. Where subgroup analyses were carried out, impact often increased with increasing intervention exposure [9], [11], [21], [22]. However, as demonstrated in this study, the measurement of exposure to interventions is a challenge especially for multi-component and community-based interventions. Most studies tend to focus on measurement of the quantity of the intervention as opposed to the quality of the intervention.

This study has shown that the desirable long-term impact of the MkV intervention on knowledge, reported attitudes and selected reported behaviours did not vary greatly according to age, marital status or time since last exposure to the intervention. From a programmatic perspective, this suggests that the intervention can have an impact on a broad cross-section of the population of young people in rural Mwanza. There was some evidence of differential impact according to the number of years exposure during the initial phase of intensively supported implementation, reinforcing the view that intervention impact can often depend on the intensity and quality of intervention delivery[23]. Intervention implementers should take steps to ensure the maintenance of intervention quality such as supervision and retraining of teachers and especially training of new teachers if teachers are transferred out. The clear dose-response findings from the initial follow-up survey [9] and some evidence of a similar pattern from the more recent follow-up survey suggest that reducing the intensity and duration of the MkV intervention may decrease the beneficial impact of the intervention. The development of effective prevention interventions is essential if rates of HIV are to continue to decrease among young people. An increased focus, within intervention evaluations, on measurement of, not only the quantity but also the quality of interventions, will improve our understanding of their effectiveness.
